# Retraction: XEDAR inhibits the proliferation and induces apoptosis of gastric cancer cells by regulating JNK signaling pathway

**DOI:** 10.1042/BSR-2019-2726_RET

**Published:** 2024-08-09

**Keywords:** Apoptosis, Gastiric cancer, JNK signaling pathway, p53, XEDAR

This article “XEDAR inhibits the proliferation and induces apoptosis of gastric cancer cells by regulating JNK signaling pathway” (DOI: 10.1042/BSR20192726) is being retracted from *Bioscience Reports* at the request of the authors, who state they have been unable to replicate the results in this paper, and therefore feel that the conclusions are affected. The authors also identified that Figure 1B GAPDH panel and Figure 3B GAPDH panel were unintentionally duplicated. Additionally, the Editorial Office identified that Figure 3B XEDAR panel seems to appear as Figure 3E FAK panel. The authors were asked to agree to the retraction statement, but did not respond. The Editor-in-Chief and Editorial Board agree with the retraction.

